# Adipose tissue derived stem cells: *in vitro* and *in vivo* analysis of a standard and three commercially available cell-assisted lipotransfer techniques

**DOI:** 10.1186/scrt536

**Published:** 2015-01-05

**Authors:** Rossana Domenis, Lara Lazzaro, Sarah Calabrese, Damiano Mangoni, Annarita Gallelli, Evgenia Bourkoula, Ivana Manini, Natascha Bergamin, Barbara Toffoletto, Carlo A Beltrami, Antonio P Beltrami, Daniela Cesselli, Pier Camillo Parodi

**Affiliations:** Department of Medical and Biological Sciences, University of Udine, P.le Kolbe 4, 33100 Udine, Italy; Clinic of Plastic and Reconstructive Surgery of Udine, University of Udine, P.le Kolbe 4, 33100 Udine, Italy; Azienda Ospedaliero-Universitaria of Udine, P.le S. Maria della Misericordia 15, 33100 Udine, Italy

## Abstract

**Introduction:**

Autologous fat grafting is commonly used to correct soft-tissue contour deformities. However, results are impaired by a variable and unpredictable resorption rate. Autologous adipose-derived stromal cells in combination with lipoinjection (cell-assisted lipotransfer) seem to favor a long-term persistence of fat grafts, thus fostering the development of devices to be used in the operating room at the point of care, to isolate the stromal vascular fraction (SVF) and produce SVF-enhanced fat grafts with safe and standardized protocols. Focusing on patients undergoing breast reconstruction by lipostructure, we analyzed a standard technique, a modification of the Coleman’s procedure, and three different commercially available devices (Lipokit, Cytori, Fastem), in terms of 1) ability to enrich fat grafts in stem cells and 2) clinical outcome at 6 and 12 months.

**Methods:**

To evaluate the ability to enrich stem cells, we compared, for each patient (n = 20), the standard lipoaspirate with the respective stem cell-enriched one, analyzing yield, immunophenotype and colony-forming capacity of the SVF cells as well as immunophenotype, clonogenicity and multipotency of the obtained adipose stem cells (ASCs). Regarding the clinical outcome, we compared, by ultrasonography imaging, changes at 6 and 12 months in the subcutaneous thickness of patients treated with stem-cell enriched (n = 14) and standard lipoaspirates (n = 16).

**Results:**

Both methods relying on the enzymatic isolation of primitive cells led to significant increase in the frequency, in the fat grafts, of SVF cells as well as of clonogenic and multipotent ASCs, while the enrichment was less prominent for the device based on the mechanical isolation of the SVF. From a clinical point of view, patients treated with SVF-enhanced fat grafts demonstrated, at six months, a significant superior gain of thickness of both the central and superior-medial quadrants with respect to patients treated with standard lipotransfer. In the median-median quadrant the effect was still persistent at 12 months, confirming an advantage of lipotransfer technique in enriching improving long-term fat grafts.

**Conclusions:**

This comparative study, based on reproducible biological and clinical parameters and endpoints, showed an advantage of lipotransfer technique in enriching fat grafts in stem cells and in favoring, clinically, long-term fat grafts.

**Electronic supplementary material:**

The online version of this article (doi:10.1186/scrt536) contains supplementary material, which is available to authorized users.

## Introduction

Fat grafting is a widely used technique that has been developed by plastic reconstructive and aesthetic surgeons [1, 2]. Adipose tissue has been used for many years as autologous filler in breast augmentation, hemifacial atrophy, facial rejuvenation and rhinoplasty [3]. However, the potential regenerative effect of fat grafting has been hypothesized and, to some extent, experimented only in recent years, as in the treatment of tissues damaged by radiotherapy [4]. The employment of adipose tissue, both as autologous filler and as a source of adipose stem cells (ASCs), has introduced the concept of regenerative therapy into plastic reconstructive surgery, thus leading to the publication of a large body of literature that supports the regenerative properties of the fat grafting techniques. ASCs are indeed mesenchymal stem cells characterized by the ability to differentiate, at least *in vitro*, into multiple cell types of all three germ layers, including adipocytes, smooth muscle cells, endothelial cells, neurons and hepatocytes [5–11].

Despite the reported promising results, however, lipofilling is burdened by variable and unpredictable graft-take rates in the postoperative period, with reported absorption rates varying from 20 to 90% [12].

The variability in fat graft survival has been attributed to several causes, such as the local angiogenesis, the donor site, sampling and processing techniques, and the type of receiving area and its characteristics. Summarizing, three main theories have been reported that try to explain the evolution of the grafted adipose tissue: Neuhof in 1923 advanced the host-replacement theory [13]; Peer in 1956 outlined the importance of adipose cell survival [14]; and Yoshimura and colleagues in 2009 demonstrated the primary role played by ASCs on the rate of fat engraftment [15]. According to this latter theory, after the lipofilling procedure, almost all of the injected cells die within a few months and are replaced by differentiation of the mesenchymal stem cells contained in the stromal vascular fraction (SVF) [16], the portion of the adipose tissue particularly enriched in ASCs [11].

For this reason, it has been proposed that the enrichment of adipose tissue with ASCs could contribute to a *de novo* formation of adipocytes and blood vessels, thus promoting long-term volume retention of the autologous fat graft. Consequently, a new approach named cell-assisted lipotransfer (CAL) has been introduced [17]. CAL consists of freshly isolating SVF cells from one-half of the aspirated fat and recombining the isolated cells with the other half, thus using, for reconstructive purposes, autologous primitive cells in combination with lipoinjection. This process allows converting relatively ASC-poor aspirated fat to ASC-rich fat [17].

Consistently, in the last years, numerous devices intended for clinical use have been developed and commercialized. These devices are aimed at increasing the concentration of primitive cells in the fat graft, by automating and standardizing the procedures for extracting, washing and concentrating the SVF from the autologous adipose tissue, thus ensuring safety and reproducibility of the procedures [3, 15, 18–23].

Although some of these clinical devices, often relying on different principles, have been described in a number of scientific reports specifically focused on the effectiveness and safety of the new proposed technologies, very few papers have directly compared these different procedures in terms of SVF and ASC enrichment as well as clinical results [18, 24]. In fact, if the long-term volume retention of the autologous fat graft is guaranteed by the presence of ASCs, it has to be demonstrated that these cells are more abundant in the enriched-adipose tissues and that they are able to persistently proliferate and differentiate into adipocytes and vessels.

In this study we analyzed four different lipotransfer techniques used for breast reconstruction: the gold-standard technique used in our department, which is a modified Coleman’s procedure, and three different devices available on the market – the Cytori Celution System (Cytori Ltd., Deeside, UK), the Lipokit Medikhan System (Medikan International Inc, Pusan, Korea) and the Fastem Corios (CORIOS Soc. Coop, San Giuliano Milanese (MI), Italy). Specifically, we focused our attention on the ASCs isolated from the different products, studying stem cell yield, proliferation and differentiation ability. This paper has: optimized an *in vitro* protocol to compare, for each patient, standard fat grafts and stem cell-enriched fat grafts, in terms of SVF cells and ASCs; demonstrated the effectiveness of the enzymatic stem cell enrichment procedures in enriching fat grafts in primitive cells; and presented preliminary clinical data showing that the use of adipose tissue enriched in stem cells seems to correlate with a better persistence of the fat graft at 6 and 12 months.

## Materials and methods

The independent ethics committee of the Azienda Ospedaliero – Universitaria of Udine approved the research (Parere N. 103/2011/Sper). Written informed consent was obtained from patients and all clinical investigations have been conducted according to the principles expressed in the Declaration of Helsinki.

### Tissue donors

We enrolled in the study 36 patients who underwent mammary reconstruction by different lipostructure techniques from January 2010 to January 2012. Four skilled senior plastic surgeons, who belong to our department, performed the surgical procedures following a standardized procedural protocol.

Table 1 summarizes the clinical features of the enrolled patients. Specifically, 16 patients underwent lipostructure with the standard technique, a Coleman’s modified procedure optimized in our department, while in 20 patients the lipostructure was obtained using autologous adipose tissue enriched in SVF through three different devices: the Cytori Celution System (Cytori [25], *n* = 9), the Lipokit Medikhan System (Lipokit [26], *n* = 5) and the Fastem Corios (Fastem, *n* = 6), following the manufacturers’ instruction. Briefly, in the standard procedure the breast reconstruction was performed using directly the centrifuged lipoaspirate. In the other cases, one-half of the lipoaspirates was employed to enzymatically (Cytori and Lipokit) or mechanically (Fastem) isolate the SVF cells and these were then added to the other half of the lipoaspirate. Lipostructure was then performed using the SVF-enriched lipoaspirate. A detailed description of the techniques is presented in Additional file 1.

**Table 1:** **Patients included in the study**

	Standard lipoaspirate	ASC-enriched lipoaspirate	Statistical significance	ASC-enriched lipoaspirate technique		
				Cytori	Lipokit	Fastem
**Clinical features**						
*n* (%)	16 (44.4%)	20 (55%)		9 (25%)	5 (13.8%)	6 (16.7%)
Age (years)	49.5 (21 to 71)	48 (19 to 74)	*P* = 0.8370	46 (41 to 70)	52 (41 to 57)	52 (19 to 74)
BMI (kg/m^2^)	22.35 (19.36 to 30.3)	21.4 (19.8 to 32.8)	*P* = 0.7520	21.30 (20.58 to 30.5)	22.19 (20 to 32.8)	21.20 (19.8 to 24.4)
Co-morbidities	0 (0.0%)	2 (10.0%)	*P* = 0.4921	1 (11.1%)	1 (20.0%)	0 (0.0%)
**Previous surgical procedure**						
Mastectomy	14 (87.50%)	16 (80.00%)	*P* = 0.6722	8 (89%)	5 (100%)	3 (50%)
Quadrantectomy	1 (6.25%)	2 (10.00%)	*P* = 1.0000	1 (11.1%)	0 (0.00%)	1 (16.7%)
None (congenital malformation)	1 (6.25%)	2 (10.00%)	*P* = 1.0000	0 (0.00%)	0 (0.00%)	2 (33.3%)
**Donor area**						
Abdomen	5 (31.25%)	1 (5.00%)	*P* = 0.0689	0 (0.00%)	0 (0.00%)	1 (16.7%)
Hips	7 (43.75%)	7 (35.00%)	chi-q = 0.5926	3 (22.2%)	3 (60%)	1 (16.7%)
Trochanteric region	2 (12.50%)	2 (10.00%)	*P* = 1.0000	1 (11.1%)	0 (0.00%)	1 (16.7%)
Abdomen and hips	2 (12.50%)	4 (20%)	*P* = 0.6722	1 (11.1%)	2 (40%)	1 (16.7%)
Hips and trochanteric region	0 (0.00%)	4 (20%)	*P* = 0.1131	3 (22.2%)	0 (0.00%)	1 (16.7%)
Abdomen and trochanteric region	0 (0.00%)	1 (5.00%)	*P* = 1.0000	0 (0.00%)	0 (0.00%)	1 (16.7%)
Abdomen. hips and trochanteric region	0 (0.00%)	1 (5.00%)	*P* = 1.0000	1 (11.1%)	0 (0.00%)	0 (0.00%)
Amount of solution infiltrated (cm^3^)	95 (54 to 160)	143 (60 to 230)	*P* = 0.0540	158 (75 to 230)	145 (60 to 175)	85 (75 to 165)
**Adjuvant therapies**						
None	6 (37.5%)	11 (55%)	*P* = 0.3351	5 (55.6%)	2 (40%)	4 (66.7%)
RT	1 (6.25%)	3 (15.00%)	*P* = 0.6129	1 (11.1%)	1 (20%)	1 (16.7%)
CT	7 (43.75%)	3 (15.00%)	*P* = 0.0732	1 (11.1%)	2 (40%)	0 (0.00%)
RT and CT	2 (12.50%)	3 (15.00%)	*P* = 1.0000	2 (22.2%)	0 (0.00%)	1 (16.7%)
Hormonal therapies	7 (43.75%)	12 (60.00%)	chi-q = 0.3318	6 (67%)	4 (80%)	2 (33.3%)

Data presented as median (range) or *n* (%). ASC, adipose stem cell; BMI, body mass index; CT, chemotherapy; Cytori, Cytori Celution System (Cytori Ltd., Deeside, UK) [25]; Fastem, Fastem Corios (CORIOS Soc. Coop, San Giuliano Milanese (MI), Italy); Lipokit, Lipokit Medikhan System (Medikan International Inc, Pusan, Korea) [26]; RT, radiotherapy.

### Stem cell isolation and culture

SVFs were isolated from aliquots of the lipoaspirates used for breast reconstruction. In the case of patients undergoing reconstruction with stem cell-enriched samples, stem cell isolation was performed from aliquots of either non-enriched adipose tissue samples (that is, collected before stem cell enrichment procedures) or stem cell-enriched adipose tissue samples.

Lipoaspirates were enzymatically dissociated using a 0.025% collagenase II solution for 30 minutes at 37°C (Worthington, Lakewood, NJ, USA) and, after neutralization of the enzyme, were centrifuged at 600 × *g* for 10 minutes and filtered (Merck Millipore, Darmstadt, Germany). To determine the cell yield after isolation, cells less than 70 μm in diameter were counted and recovery was expressed as 10^6^ cells per milliliter of lipoaspirate.

To isolate and culture ASCs, we applied, with minor modifications, a protocol optimized for culturing multipotent adult stem cells from normal human tissues [5–7, 9, 27, 28] and neoplastic human tissues [29, 30]. Briefly, freshly isolated SVF cells (1.5 × 10^6^) were plated onto human fibronectin-coated (Sigma-Aldrich, St. Louis, MO, USA) 100 mm dishes (Corning BV Life Sciences, Corning, NY, USA) in a proliferation medium composed as follows: 60% low glucose Dulbecco’s modified Eagle’s medium (Invitrogen Life Technologies, Carlsbad, CA, USA), 40% MCDB-201, 1 mg/ml linoleic acid–bovine serum albumin, 10^-9^ M dexamethasone, 10^-4^ M ascorbic acid-2 phosphate, 1× insulin–transferrin–sodium selenite (all from Sigma-Aldrich), 2% fetal bovine serum (StemCell Technologies, Grenoble, France), 10 ng/ml human platelet-derived growth factor-BB, 10 ng/ml human epidermal growth factor (both from Peprotech EC, London, UK) [5–7, 9, 27, 28]. Medium was replaced with fresh medium every 4 days. Once cells reached 70 to 80% confluence, they were detached by TrypLE Express (Invitrogen) and replated at a density of 1 × 10^3^/cm^2^ to 2 × 10^3^/cm^2^.

### Cell culture assays

Colony-forming efficiency, cell growth kinetics, single-cell cloning and multilineage differentiation were performed as descried previously [6, 7, 9, 27].

Colony-forming units of SVF cells were determined by seeding freshly isolated cells at a concentration of 1,500 cells/cm^2^ in 96-well plates and counting the number of formed colonies under a phase contrast microscope 7 days after incubation at 37°C. Colony-forming units were examined in triplicate and by two independent investigators.

The cell population doubling time was calculated by plating ASCs in the third passage at a density of 2 × 10^3^ cells/cm^2^ in expansion medium. Medium was replaced with fresh medium every 4 days and, at different time points (1, 2, 3, 5 and 7 days), cells were detached with TrypLE Express (Invitrogen) and counted. The population doubling time was calculated during the log phase of growth.

To generate single cell-derived clones, a cell sorter (MoFlo; Beckman Coulter s.r.l., Milan, Italy) was used to automatically deposit individual ASCs onto fibronectin-coated wells of 96-well Terasaki plates (*n* = 300 wells/cell line). The cells were cultured in expansion medium with 10% fetal bovine serum. To determine the sorting efficiency and to verify whether any well was properly seeded, we utilized Vybrant CFDA SE as a cell tracker (Molecular Probes, Invitrogen). Wells were examined once a week. When clones reached confluence, cells were detached utilizing TrypLE (Invitrogen) and plated at the density of 1.5 × 10^3^cells/cm^2^ on new fibronectin-coated dishes and cultured in expansion medium.

ASCs were differentiated and analyzed as described previously [6, 7, 9, 27]. Hepatocytic differentiation was induced by growing cells for 2 weeks at high density (2 × 10^4^/cm^2^) onto fibronectin-coated coverslips in a medium containing 0.5% fetal bovine serum (Sigma-Aldrich), 10 ng/ml fibroblast growth factor-4 and 20 ng/ml hepatocyte growth factor (both from Peprotech EC). Muscle cell and endothelial cell differentiation was achieved by plating 0.5 × 10^4^/cm^2^ to 1 × 10^4^/cm^2^ cells in expansion medium containing 5% fetal bovine serum (Sigma-Aldrich), 10 ng/ml basic fibroblast growth factor, 10 ng/ml vascular endothelial growth factor and 10 ng/ml insulin-like growth factor-1 (all from Peprotech EC), but not epidermal growth factor. Adipogenic differentiation was induced using culture medium supplemented with 0.5 mM isobutylmethylxanthine, 50 μM indomethacin and 0.5 μM dexamethasone. Cells were allowed to become confluent and cultured for up to 2 weeks with medium exchanges every 4 days.

### Flow cytometry

Freshly isolated cells or ASCs at the third passage in culture were detached by Tryple Express and were incubated with the properly conjugated antibodies CD10, CD13, CD29, CD34, CD44, CD45, CD49a, CD49b, CD49d, CD59 (BD Biosciences, Milan, Italy), CD66e (AbD Serotec, Kidlington, UK), CD73, CD90 (BD Biosciences), CD105 (Serotec), CD117 (BD Biosciences), CD133 (Miltenyi Biotec, Bergisch Gladbach, Germany), CD271 (R&D Systems, Minneapolis, MN, USA), HLA-DR (BD Biosciences), KDR and ABCG-2 (R&D System). Properly conjugated isotype-matched antibodies were used as a negative control. The analysis was performed either by FACS-Calibur or by FACSCanto (BD Bioscience) [6, 7, 9, 27]. Erythrocytes, in freshly isolated samples, were lysed by incubating cells for 5 minutes in fluorescence-activated cell sorting lysing solution (BD Bioscience).

### Immunofluorescence and cytochemistry

Cell staining and image acquisition were conducted as described previously [27, 28] to detect Oct-4 (1:150; Abcam, Cambridge, UK), Sox-2 (1:150; Chemicon International Inc., Billerica, MA, USA), Nanog (1:150; Abcam), Nestin (1:100; Millipore), vimentin (1:100; Dako, Glostrup, Denmark), peroxisome proliferator-activated receptor gamma (1:100; Santa Cruz Biotechnology. Inc., Heidelberg, Germany), CD31 (1:50; Dako), smooth muscle actin (1:50; Dako), alpha sarcomeric actin (1:100; Sigma) and c-Kit (1:30; Dako). The accumulation of lipid droplets indicating adipogenic differentiation was detected staining the cells in a solution of 0.5% Oil Red-O (Sigma-Aldrich).

### Clinical follow-up

To compare the clinical results of the different lipostructure techniques used in the mammary reconstruction, ultrasound measurement of the subcutaneous thickness in the reconstructed breast have been acquired preoperatively as well as 6 and 12 months postoperatively. Moreover, representative preoperative and postoperative photographs of the mammary region, taken according to standard projections, have been acquired. Ultrasound measurements (7.5/13.5 MHz) have been collected with the GE Vivide portable ultrasound Doppler (GE Healthcare, Milan, Italy). In order to standardize the procedure, the breast mound has been divided into nine quadrants and, although measurements were performed on all the quadrants, comparisons between groups were performed on the superior-medial quadrant (SMQ) and on the median-median quadrant (MMQ; that is, the central quadrant in the breast mound). In fact, as reported in the literature [31], both the SMQ and the MMQ represent an area of weakness and thinness in the reconstructed breast after mastectomy. The investigated area corresponds to the treated area in the patients who underwent quadrantectomy reconstructions and congenital malformation treatment.

Depending on the observational nature of the study and the impossibility for some patients to undergo preoperative or postoperative ultrasound, some data regarding presurgery or postsurgery quadrant subcutaneous thicknesses were incomplete. Thickness gain data were thus not available for seven patients. Analyses were performed without imputing for missing data.

### Statistical analysis

For the ASC study, characteristics of the population have been described using the mean ± standard deviation or median and range for continuous variables and percentages for categorical variables. Data were tested for normal distribution using the Kolmogorov–Smirnov test. Paired *t* test or Mann–Whitney test, as appropriate, was used to compare continuous variables between two groups. Repeated-measurements one-way analysis of variance followed by the Bonferroni post test or the Kruskal–Wallis test followed by Dunn’s test were used, as appropriate, to compare more than two groups. *P* <0.05 was considered significant (Prism, version 4.0c; GraphPad Software, Inc., La Jolla, CA USA).

For the clinical study, patient characteristics were described using the median and range for continuous variables, and frequencies and percentages for categorical variables. A *t* test or Wilcoxon–Mann–Whitney test was used to compare continuous variables between standard treatment and stem cell-enriched treatment, in accordance with Shapiro–Wilk’s normality test results. The chi-square test or Fisher exact test was used to compare categorical variables, as appropriate. For detailed comparisons among the standard group and the three enrichment methods, the Kruskall–Wallis test was performed because of the small sample sizes. Preoperative and long-term data were tested with the Wilcoxon signed rank-sum test, while the Friedman test were used to compare three groups of data.

Analyses were performed without imputing for missing data. The total number of subjects analyzed is reported.

## Results

### Patients enrolled in the study

As summarized in Table 1, most of the patients enrolled in the study underwent breast reconstruction after mastectomy (*n* = 30), while a minority of them was treated after a quadrantectomy (*n* = 3) or for a congenital malformation (Poland syndrome, *n* = 3). Patients treated with the standard method did not differ significantly from those treated with the stem cell-enriched procedure in terms of age, body mass index, co-morbidities and therapies.

Lipoaspirates were obtained in most cases from the hip area, followed by the abdominal and the trochanteric regions, without significant differences between patients treated either with the standard or ASC enrichment procedure.

### Isolation of adipose tissue stem cells from lipoaspirates obtained by different procedures

**Figure 1:**
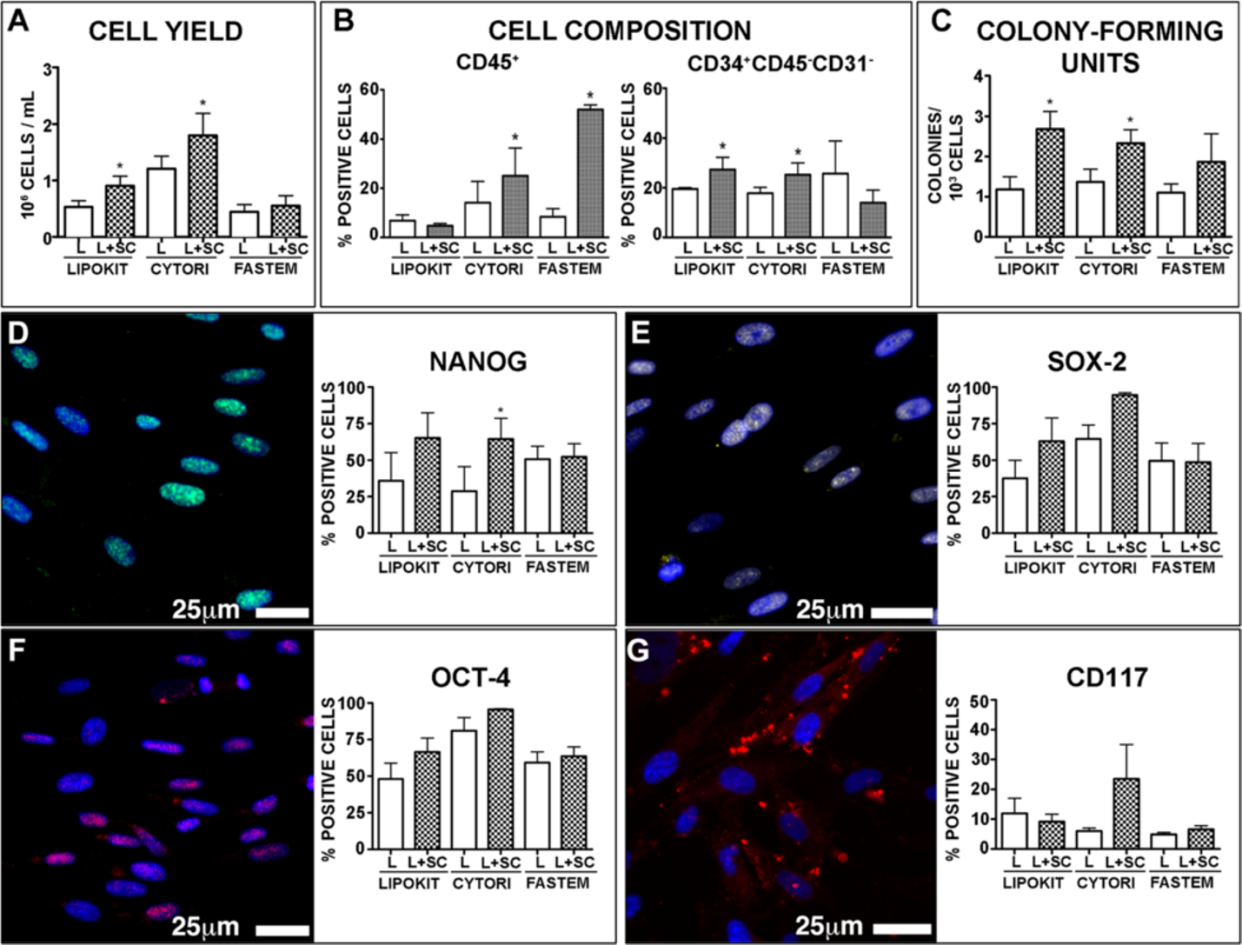
**Isolation of primitive cells from lipoaspirates and lipoaspirates enriched with stem cells.** Histograms represent **(A)** cell yield, **(B)** cell composition and **(C)** number of colony-forming units at 7 days from seeding. **(D, E, F, G)** Expression of stem cell markers in primary culture. Proliferating cells expressed high levels of Nanog (green fluorescence, **D**), Sox-2 (yellow fluorescence, **E**) and Oct-4 (magenta fluorescence, **F**), while a fraction of cells expressed c-Kit (red fluorescence, **G**). Nuclei are depicted by the blue fluorescence of 4′,6-diamidino-2-phenylindole. In the histograms, results are expressed as mean ± standard deviation. For each isolation method, *P <0.05 vs. lipoaspirates. Cytori, Cytori Celution System (Cytori Ltd., Deeside, UK) [25]; Fastem, Fastem Corios (CORIOS Soc. Coop, San Giuliano Milanese (MI), Italy); L, lipoaspirates; L + SC, stem cell-enriched lipoaspirates; Lipokit, Lipokit Medikhan System (Medikan International Inc, Pusan, Korea) [26].

To establish whether the enrichment procedures could interfere with the ability to isolate primitive cells from lipoaspirates, we analyzed 40 lipoaspirates obtained from 20 patients that underwent a CAL procedure. Specifically, for each patient we compared the characteristics of freshly isolated cells both from the non-enriched samples (*n* = 20) and the stem cell-enriched samples (*n* = 20).We observed that, at the end of the enzymatic dissociation followed by the filtration step, both the Cytori and Lipokit procedures determined a significant increase in the number of recovered cells, when compared with the respective non-enriched lipoaspirates. This was not the case for the Fastem procedure (Figure 1A).

To establish whether the enrichment procedure modified the composition of the isolated cells, we performed a fluorescence-activated cell sorting analysis to determine, in the erythrocyte-lysed samples, the fraction of CD45^+^ hematopoietic cells and, among the CD45-negative cells, the expression of the mesenchymal stem cell markers CD73 and CD90 and of the endothelial marker CD31. Moreover, we evaluated the CD34^+^CD45^-^CD31^-^ stromal cell population [32]. The Cytori and, especially, Fastem procedures significantly increased the fraction of CD45-positive hematopoietic cells, while the CD34^+^CD45^-^CD31^-^ stromal cell population was significantly increased by both the Lipokit and Cytori enrichment procedures (Figure 1B). No main differences were detected in the frequency of CD31-positive, CD73-positive or CD90-positive cells.

When dissociated cells were cultured to obtain ASCs, only a minority of the seeded cells was able to adhere and proliferate (Figure 1C). Specifically, the colony-forming efficiency, an assay often used to quantify the presence of mesenchymal stem cells [33], was significantly increased in samples enriched by the two enzymatic methods, Cytori and Lipokit, but not by the mechanic Fastem method (Figure 1C).

Despite the stringent culture conditions, proliferating cell lines were obtained from every sample 5 to 7 days after the primary culture confirming the high efficiency of the optimized method [5–7, 9, 27].As soon as 1 week after seeding, proliferating cells were positive for the expression of the pluripotent state-specific transcription factors Oct-4, Nanog and Sox-2 (Figure 1D,E,F), thus excluding that the acquisition of these features could be related to an extensive culture manipulation. The expression of c-Kit, the stem cell antigen receptor, was detected in about 10% of the population without significant differences between the samples (Figure 1G).

Altogether, these results indicate that samples obtained by the Cytori and Lipokit stem cell enrichment procedures are characterized, with respect to the non-enriched counterpart, by increased cell yield and number of colony-forming cells. Conversely, the Fastem procedure seems not to affect these parameters.

### Cytori and Lipokit enrichment in stem cells persists with passages in culture

To test the hypothesis that the persistence of ASCs in the donated fat is required for the long-term volume retention of the autologous fat graft, we verified whether the enrichment in primitive cells persisted after extensive expansion in culture. For this purpose, ASCs obtained from samples enriched with the three different techniques were compared with those isolated from their nonstem cell-enriched counterparts.

**Figure 2:**
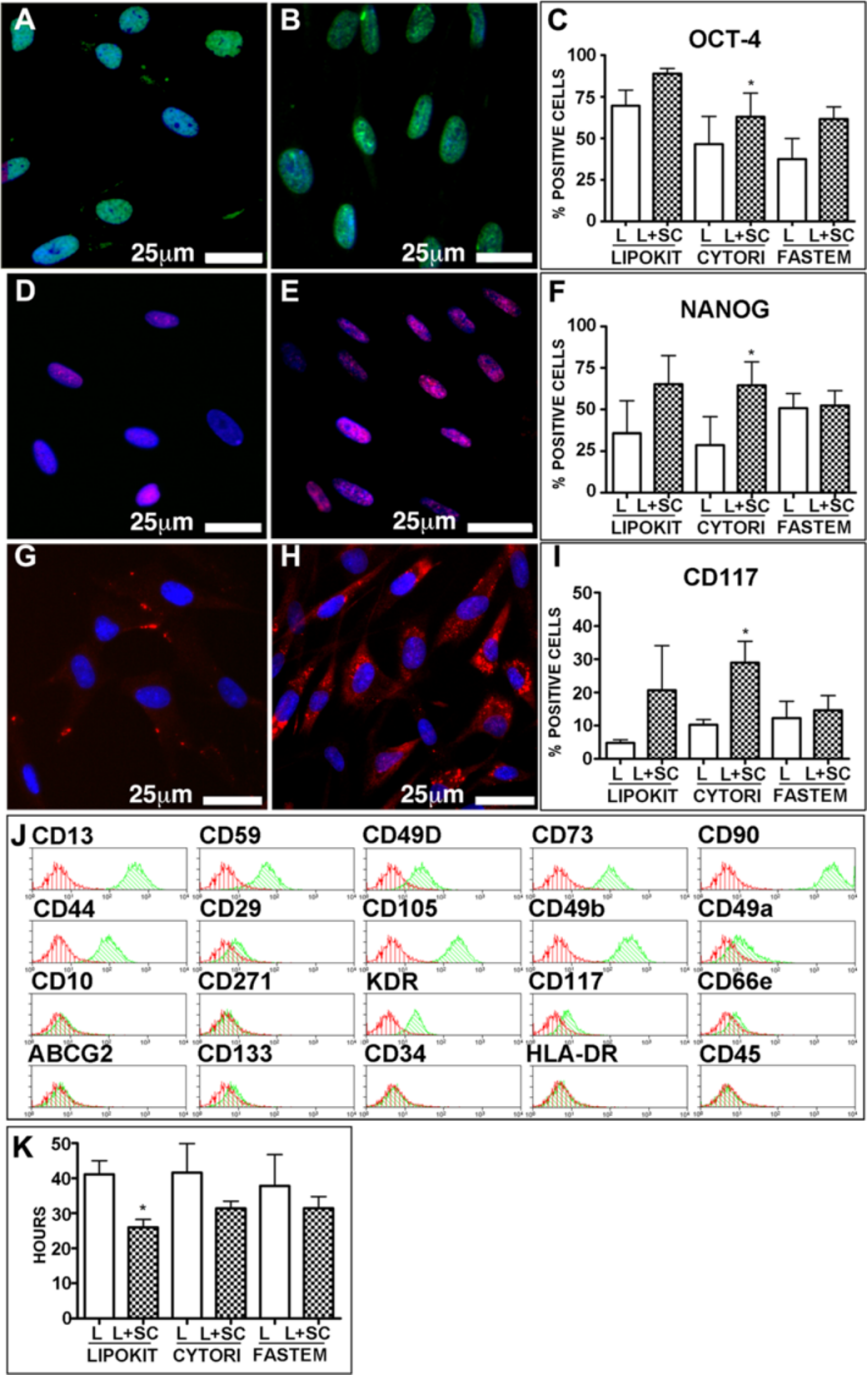
**Stem cell marker expression by adipose stem cells.** Stem cells isolated from lipoaspirates (L) **(A, D, G)** and from lipoaspirates enriched with stem cells (L + SC) **(B, E, H)** express Oct-4 (green fluorescence, **A**, **B**, **C**), Nanog (magenta fluorescence, **D**, **E**, **F**) and c-Kit (red fluorescence, **G**, **H**, **I**). Nuclei are depicted by the blue fluorescence of 4′,6-diamidino-2-phenylindole. **(J)** Representative surface immunophenotype of adipose stem cells: histogram overlay shows isotype control IgG staining profile (red histograms) versus specific antibody staining profile (green histograms). **(K)** Population doubling time. (**C**, **F**, **I**, **K**) Results expressed as mean ± standard deviation. *P <0.05 vs. L. Cytori, Cytori Celution System (Cytori Ltd., Deeside, UK) [25]; Fastem, Fastem Corios (CORIOS Soc. Coop, San Giuliano Milanese (MI), Italy); Lipokit, Lipokit Medikhan System (Medikan International Inc, Pusan, Korea) [26].

As shown in Figure 2, proliferating cells at the third passage in culture (that is, ≈24th population doubling) continued to express Oct-4 and Nanog (Figure 2A,B,C,D,E,F), transcription factors considered crucial, together with Klf-4 and c-Myc, for the maintenance of a primitive state [34]. However, only Cytori enriched samples were characterized, with respect to the non-enriched counterpart, by a significant increase in the expression of both Oct-4 and Nanog (Figure 2C,F). Furthermore, 20 to 30% of cultured cells derived from samples enriched in stem cells by either Lipokit or Cytori expressed the stem cell marker c-Kit, although only the latter group significantly differed from the non-enriched counterpart (Figure 2G,H,I).

The flow cytometric analysis of the cell surface immunophenotype (Figure 2J) demonstrated that every cell line exhibited, independently of the enrichment procedure, a similar mesenchymal phenotype, characterized by the expression of CD44, CD73, CD90 and CD105 in the absence of hematopoietic markers, such as CD45. With the exception of c-Kit, which was more expressed both in Cytori and Lipokit enriched samples, no other major differences were detected (Additional file 2).When analyzed in terms of growth kinetic, only cell lines obtained from samples enriched in stem cells by Lipokit showed a significantly reduced population doubling time, while for Cytori enriched and Fastem enriched samples the trend in increasing the growth speed did not reach a statistical significance (Figure 2K).

**Figure 3:**
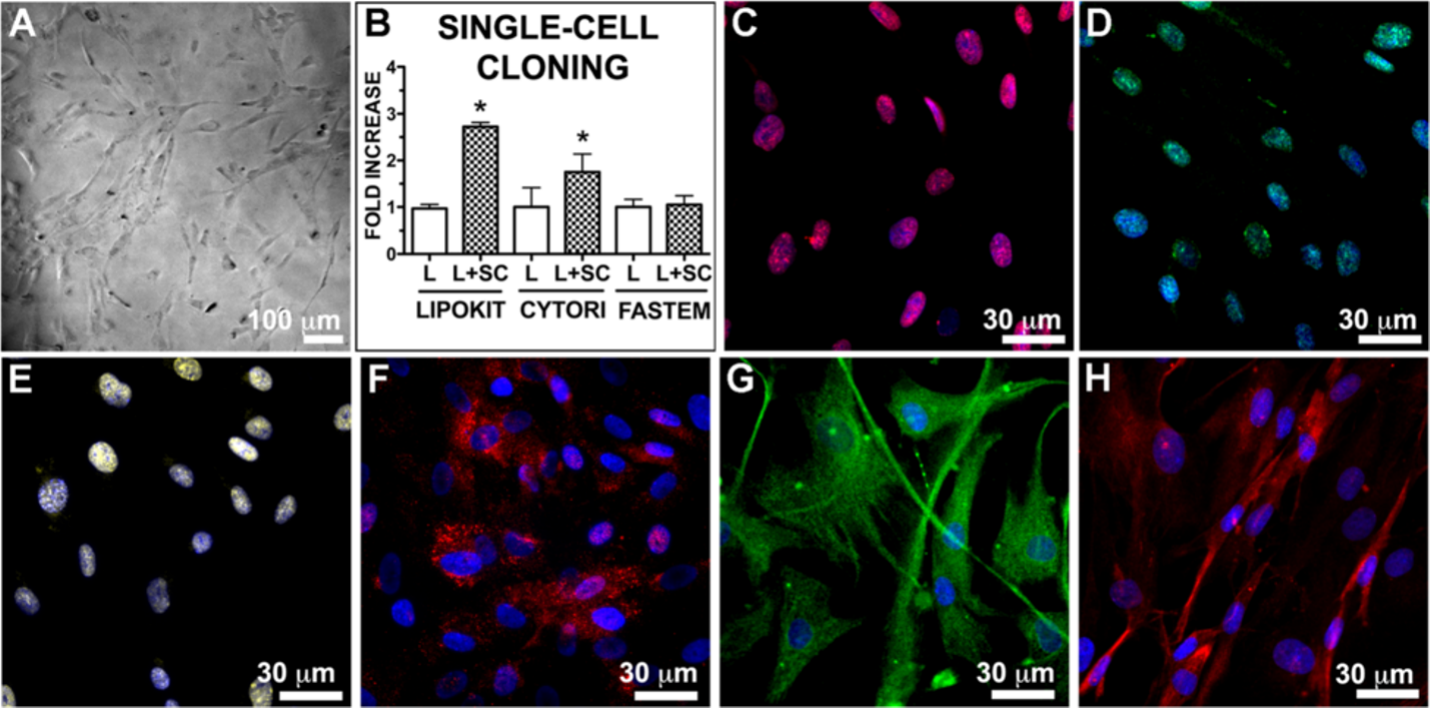
**Clonogenicity of adipose stem cells. (A)** Phase contrast image of a representative single-cell derived clone. **(B)** Quantitative evaluation of the clonogenicity of stem cells isolated from lipoaspirates (L) and from lipoaspirates enriched with stem cells (L + SC). Data expressed as mean ± standard deviation. *P <0.05 vs. L. **(C, D, E, F)** Clones are characterized by a primitive phenotype: highly expressing Oct-4 (red fluorescence, **C**), Nanog (green fluorescence, **D**), Sox-2 (yellow fluorescence, **E**), c-Kit (red fluorescence, **F**), and the intermediate filaments nestin (green fluorescence, **G**) and vimentin (red fluorescence, **H**). Cytori, Cytori Celution System (Cytori Ltd., Deeside, UK) [25]; Fastem, Fastem Corios (CORIOS Soc. Coop, San Giuliano Milanese (MI), Italy); Lipokit, Lipokit Medikhan System (Medikan International Inc, Pusan, Korea) [26].

Finally, to test whether enrichment procedures affected clonogenicity, a crucial *in vitro* property of stem cells, we performed a single cell-cloning assay comparing non-enriched and stem cell-enriched paired samples (Figure 3). Although it was possible to isolate highly proliferating clones from all cell lines (Figure 3A,B), the frequency of these clones was significantly increased in Cytori and Lipokit enriched ASCs (Figure 3B). Specifically, ASCs enriched by Lipokit were 2.8 (±0.6) times more clonogenic than the non-enriched counterpart, while Cytori enrichment determined a 2.5 (±2.1)-fold increase in the frequency of clonogenic cells. Enrichment by Fastem did not increase the clonal ability of isolated cells. Importantly, clonal cells maintained a stable undifferentiated phenotype characterized by the expression of pluripotent state-specific transcription factors vimentin, nestin and c-Kit (Figure 3C,D,E,F,G,H).

### Adipose stem cells from Lipokit and Cytori enriched fat grafts display superior differentiation potential *in vitro*

**Figure 4:**
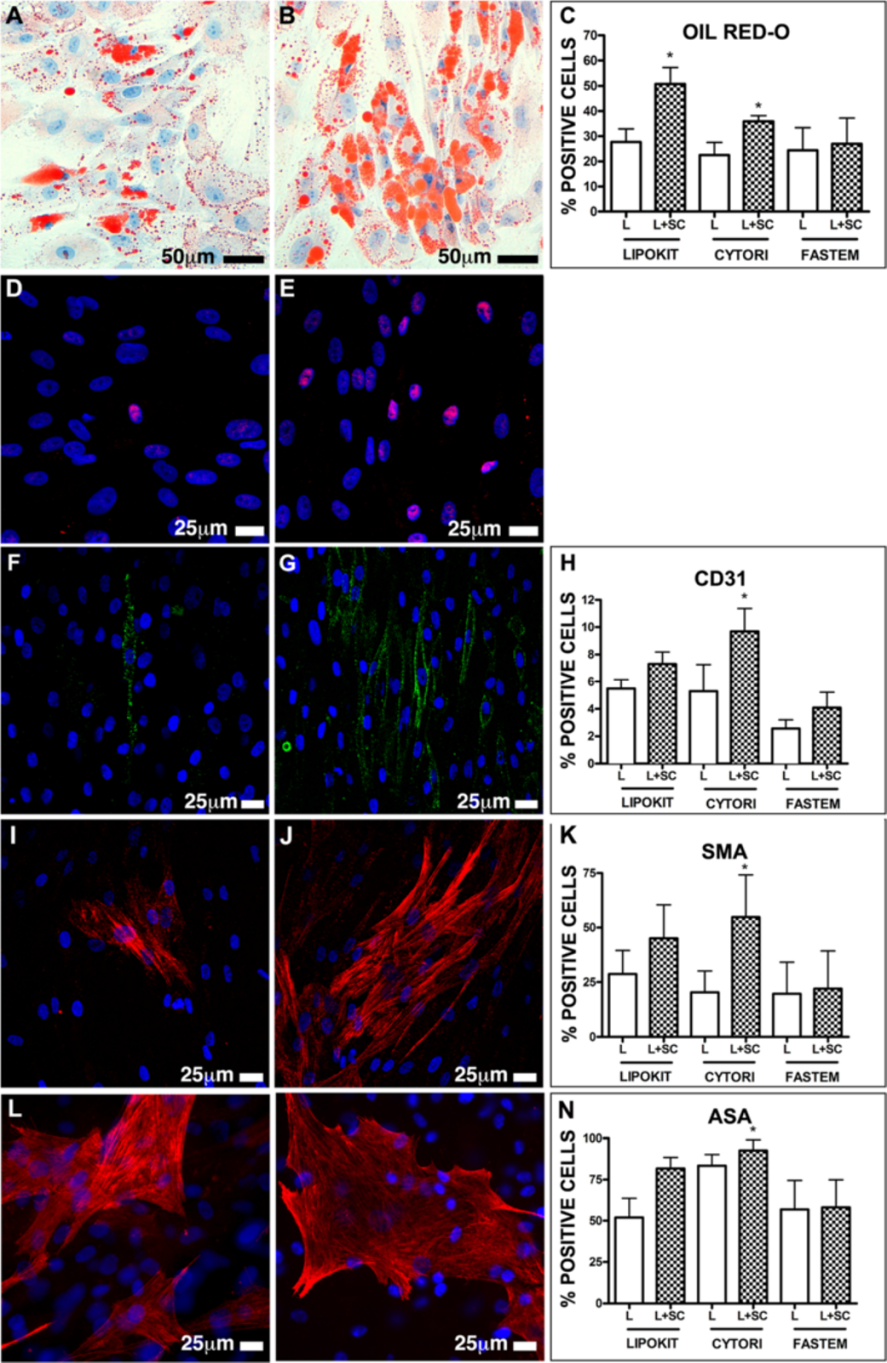
**Multipotency of adipose stem cells.** Stem cells isolated from lipoaspirates (L) **(A, D, F, I, L)** and from lipoaspirates enriched with stem cells (L + SC) **(B, E, G, J, M)** were evaluated for their ability to differentiate into adipocyte-like cells staining positive for Oil-red O (red vacuoles, **A**, **B**) and peroxisome proliferator-activated receptor gamma (magenta, **D**, **E**), endothelial-like cells positive for CD31 (green fluorescence, **F**, **G**), and muscle cells positive for smooth muscle actin (SMA; red fluorescence, **I**, **J**) and alpha sarcometic actin (ASA; red fluorescence, **L**, **M**). Nuclei are depicted by the blue staining of either hematoxylin or 4′,6-diamidino-2-phenylindole. (**C**, **H**, **K**, **N**) Results expressed as mean ± standard deviation. *P <0.05 vs. L. Cytori, Cytori Celution System (Cytori Ltd., Deeside, UK) [25]; Fastem, Fastem Corios (CORIOS Soc. Coop, San Giuliano Milanese (MI), Italy); Lipokit, Lipokit Medikhan System (Medikan International Inc, Pusan, Korea) [26].

It has been hypothesized that the enrichment of adipose tissue with stem cells could stimulate the formation of new blood vessels and maintain the thickness of the graft. In order to establish whether the enrichment procedure could increase the frequency, within grafted samples, of multipotent cells able to originate new vessels and adipose tissue, we exposed ASCs to adipogenic-inducing, myogenic-inducing and endothelial-inducing conditions (Figure 4). Cells cultured in adipocyte differentiation medium accumulated Oil Red-O-positive lipid droplets in their cytoplasm (Figure 4A,B,C) and increased the expression of peroxisome proliferator-activated receptor gamma (Figure 4D,E). Again, cell lines obtained from Lipokit and Cytori enriched samples displayed a significantly increased ability to differentiate into adipocyte-like cells.Concerning the ability to differentiate into CD31-positive endothelial cells, every tested cell line was able to acquire endothelial cell features (Figure 4F,G,H). However, only Cytori enrichment significantly improved the endothelial differentiation capacity of ASCs (Figure 4H). Similarly, only cell lines obtained from samples enriched by Cytori were characterized by an increased ability to differentiate into both smooth muscle cells (Figure 4I,J,K) and skeletal muscle cells (Figure 4L,M,N). ASCs obtained from Lipokit enriched samples displayed only a trend towards the improvement in their differentiation ability, while ASCs obtained from Fastem enriched samples did not differ from those obtained from the non-enriched counterparts.

Altogether, the accumulated evidence showed that cell lines obtained from Cytori enriched samples were characterized by an increased ability to differentiate into endothelial-like, myocyte-like and adipocyte-like cells.

### Adipose tissue grafts enriched by stem cells improve subcutaneous thickness gain

**Figure 5:**
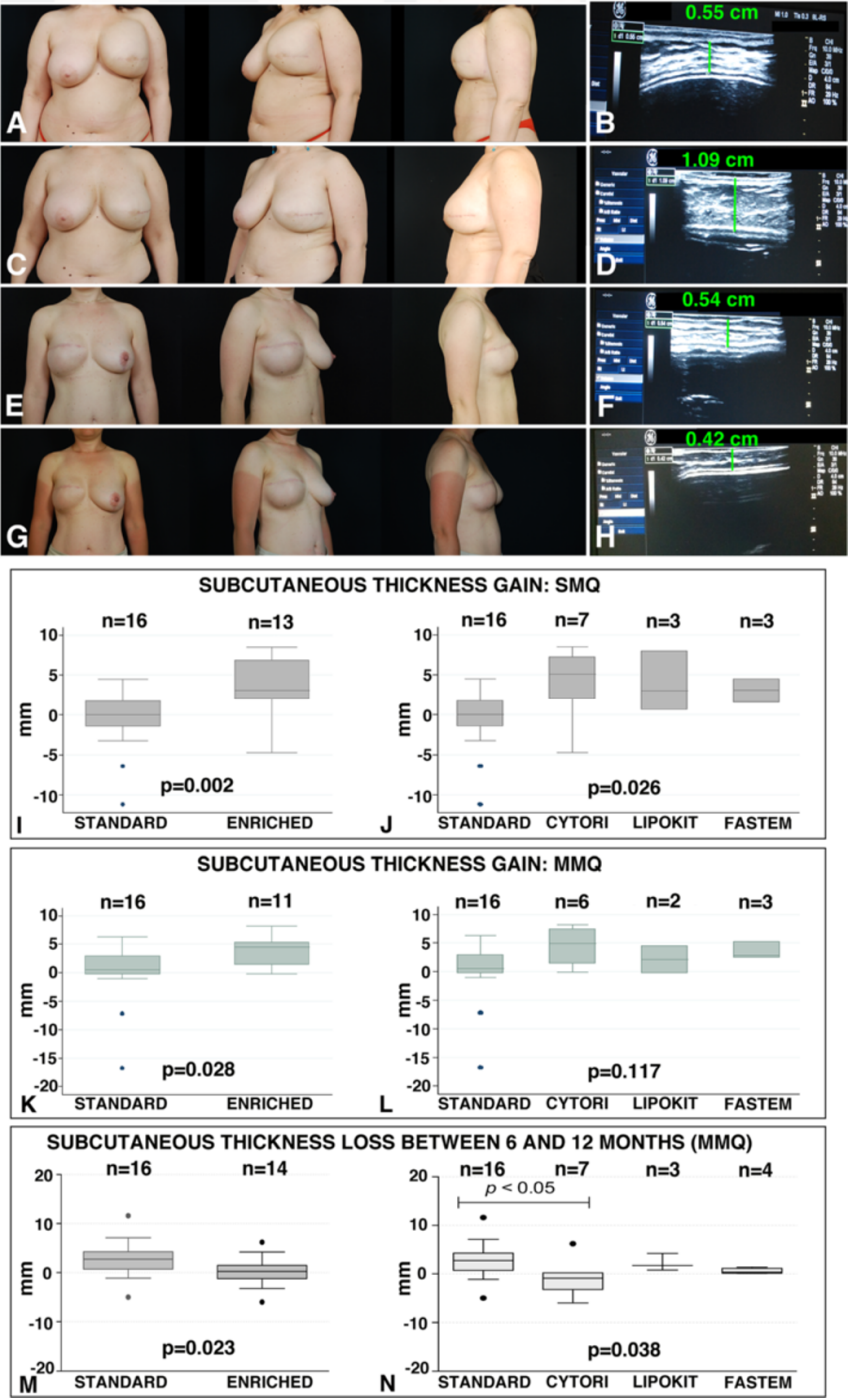
**Clinical evaluation. (A, B, C, D)** Patient treated with a stem cell-enriched fat grafting technique. **(A)** Preoperative pictures of the patient after mastectomy and immediate expander reconstruction of the left breast. (**B**) Ultrasound evaluation image of the preoperative subcutaneous thickness of the mastectomy skin flap over the implant, detected at the level of the median-median quadrant (MMQ). (**C**) Postoperative pictures at 6 months after the patient underwent expander substitution with an implant and contemporary fat grafting with 230 cm^3^ stem cell-enriched adipose tissue (Cytori). Shape, volume, and projection of the final implant are good to excellent. **(D)** Ultrasound evaluation image of the subcutaneous thickness of the mastectomy skin flap at 6 months, detected in the area of the MMQ. **(E, F, G, H)** Patient treated with the standard fat grafting technique. **(E)** Preoperative pictures of a patient after mastectomy and immediate expander reconstruction of the right breast. **(F)** Ultrasound evaluation image of the subcutaneous thickness of the mastectomy skin flap over the implant in the preoperative period, detected at the level of the MMQ. **(G)** Postoperative pictures at 6 months. Reconstruction was performed with expander substitution with an implant and contemporary fat grafting with 110 cm^3^ adipose tissue (standard technique). The symmetry is reached, although in the lateral view there is a lack in the right breast projection. **(H)** Ultrasound evaluation image of the subcutaneous thickness of the mastectomy skin flap at 6 months, detected in the area of the MMQ. **(I, J, K, L)** Graph boxes showing the subcutaneous thickness gain (mm) in the superior-median quadrant (SMQ) **(I, J)** and in the MMQ **(K, L)** at 6 months. **(M, N)** Graph boxes showing the subcutaneous thickness loss (mm) in the MMQ between 6 and 12 months. Cytori, Cytori Celution System (Cytori Ltd., Deeside, UK) [25]; Fastem, Fastem Corios (CORIOS Soc. Coop, San Giuliano Milanese (MI), Italy); Lipokit, Lipokit Medikhan System (Medikan International Inc, Pusan, Korea) [26].

To establish whether the use of stem cell-enriched lipoaspirates for breast cancer reconstruction can determine, with respect to the standard procedure, an improvement in the clinical outcome (Figure 5A,C,E,G), we compared the preoperative and postoperative subcutaneous thickness of the breast area by ultrasound measurements (Figure 5B,D,F,H), allowing us to compute the gain in subcutaneous tissue 6 months after surgery (Figure 5I,J,K,L). Importantly, the biological properties of the lipoaspirate products that were employed for the standard procedure did not differ from the above-described, nonstem cell-enriched samples (Additional files 2 and 3).

Compared with the standard technique, stem cell enrichment led to better results in terms of thickness gain in both the SMQ (+0 mm (-11.2 mm to +4.5 mm) in the standard treatment group vs. +3.1 mm (-4.7 mm to +8.5 mm) in the stem cell-enriched group, *P* = 0.002; Figure 5I) and in the MMQ (+0.5 mm (-16.6 mm to +6.3 mm) in the standard treatment group vs. +4.5 mm (-0.20 mm to +8.2 mm) in the stem cell-enriched group, *P* = 0.028; Figure 5K). When considering the single technique’s performance, a difference in thickness gain was observed for SMQ (*P* = 0.026; Figure 5J) but not for MMQ (*P* = 0.117; Figure 5L); however, subgroup analysis did not identify differences between the techniques, most probably because of small sample sizes.

Finally, to evaluate the stability of fat grafts at 1 year, we evaluated the thickness loss between 6 and 12 months in the patients treated with stem cell-enriched lipoaspirates and standard lipoaspirates, respectively. Again, compared with the standard technique, stem cell enrichment led to a significant reduction in thickness loss in the MMQ (Figure 5M), while in the SQM the difference did not reach statistical significance (data not shown). When considering the single technique’s performance, a difference in thickness gain was observed for MMQ (*P* = 0.026; Figure 5N), and the subgroup analysis identified the significant superiority of Cytori.

## Discussion

Adipose tissue has been used for a century as autologous filler. After the first attempts, explored by Neuber and Czerny at the end of the 19th century [1, 2, 34], lipofilling techniques have undergone a huge development in the 1990s thanks to Coleman’s work, rapidly gaining widespread diffusion [35, 36].

The success of this technique is strictly related to the properties of the adipose tissue, an easily accessible source that can be collected by procedures that are relatively simple, safe and repeatable with minimal morbidity. Moreover, since the adipose tissue is available in the majority of the patients, it allows autologous approaches devoid of immunogenic drawbacks. The main disadvantage of lipofilling remains the unpredictable fat graft’s resorption rate that may vary between 20 and 90% [12, 37].

Since the demonstration that adipose tissue is an abundant and accessible source of multipotent stem cells [11], the concept of stem cell-enhanced fat grafting has been formulated [17]. Briefly, the hypothesis is that the supplementation of fat grafts with cells obtained from the SVF, a cell population known to be enriched in ASCs [32], would improve graft stability, taking advantage of the capacity of ASC to reduce apoptosis, contribute to vessel formation and maintain a self-renewing reservoir of cells able to give rise to all the mature cell types constituting the adipose tissue [18, 38, 39]. In this regard, the first clinical studies of the Yoshimura group – showing the ability of the so-called CAL to improve the results of the lipostructure technique, leading to a more predictable and stable result [17, 39, 40] – were further confirmed by other groups both at clinical level [23, 41, 42] and preclinical level [38, 43–45]. Led by the accumulation of data documenting safety and efficacy of CAL approaches, different automated, semiautomated and manual devices to be used in the operating room at the point of care have been developed to isolate with safe and standardized protocols the SVF and to produce SVF-enhanced fat grafts [18, 21, 39, 46–50]. However, it is difficult for the clinician to choose among the available devices, since very few comparative studies have been performed that have taken into account systematically both biological and clinical criteria. In fact, it would be advisable to have standards based on reproducible parameters and endpoints that could allow comparing different techniques [32, 51].

In this paper, we compared a standard lipotransfer technique, based on Coleman’s procedure, with three commercially available cell lipotransfer techniques. Two of the devices, Cytori [18, 22] and Lipokit [18, 49], are closed systems allowing an enzymatic separation of the SVF cells, while the third, Fastem, is a closed system of filtering bags that mechanically isolates the SVF.

The comparison took into consideration both biological and clinical criteria. Regarding the first evaluation, we compared, for each patient, standard lipoaspirates with the respective stem cell-enriched ones in terms of yield, immunophenotype and colony-forming capacity of the SVF cells as well as immunophenotype, clonogenicity and multipotency of the culture-expanded ASCs [32]. Regarding the clinical criteria, we compared, in terms of subcutaneous thickness gain as detected by ultrasound imaging, the group of patients treated with stem cell-enriched lipoaspirates with a group of patients treated with the standard procedure.

Considering the cell yield, a higher number of cells could be isolated from lipoaspirates enriched by both of the enzymatic methods. Regarding the characterization of the isolated cells, Cytori and, especially, Fastem significantly enriched the lipoaspirate product in hematopoietic cells, while the CD34^+^CD31^-^CD45^-^ putative stromal cell-containing population [32] was higher in both Lipokit and Cytori enriched samples. A recent report by Yoshimura’s group trying to define the cellular events guiding the graft take in a murine model showed that CD34^+^ cells were proliferating in the first 2 to 4 weeks after grafting [52], while a xenograft transplantation study by Rubin’s group suggested that the concentration of CD34^+^ progenitor cells within the SVF could be used to predict the human fat graft retention [45].

Accordingly, Cytori and Lipokit enriched lipoaspirates were also characterized by an increased number of colony-forming unit cells. The colony-forming unit assay, commonly used to assess the frequency of progenitor cells [32], supported an increased number of primitive cells in Cytori and Lipokit enriched products. The trend was evident also for the Fastem procedure, although it did not reach statistical significance.

We further analyzed the stem cell-enriched lipoaspirates isolating, culturing and characterizing ASCs. Many works have been conducted showing that stem cell enrichment devices, such as Cytori, are able to isolate ASCs, thus allowing the surgeon to perform a CAL [18, 21, 22]. Additionally, recent work by Dominici’s group has demonstrated, in a rabbit subcutaneous adipose tissue autologous regeneration model, the capacity of ASCs to reduce cell apoptosis favoring a long-term graft performance [38]. More importantly, a randomized placebo-controlled trial has demonstrated that enrichment of autologous fat grafts with *ex vivo* expanded ASCs significantly improved graft survival [37].

In our study, ASCs could be isolated both from non-enriched and stem cell-enriched samples. However, differences could be detected in ASC phenotype and function at the third passage in culture. Specifically, a larger fraction of ASC obtained from Lipokit and Cytori enriched lipoaspirates expressed stem cell markers such as Nanog, Sox-2 and c-Kit, although differences reached statistical significance only for Cytori. Similarly, ASCs from the stem cell-enriched lipoaspirates tended to grow faster. Regarding clonogenicity, Lipokit and Cytori derived ASCs were characterized by a double number of cells able to grow when seeded as single cells. Similarly, ASCs obtained from the samples enriched by Lipokit and Cytori were characterized, with respect to those obtained from the non-enriched lipoaspirates, by a significantly increased ability to differentiate into adipocytes (Lipokit and Cytori), endothelial cells (Cytori) and muscle cells (Cytori). Conversely, the mechanic Fastem enrichment procedure seemed not to significantly enrich the samples in clonogenic and multipotent ASCs.

Altogether, the cytological criteria analyzed in this paper seem to indicate a clear ability of the enzymatic-based method to enrich the samples in both vascular stromal cells and ASCs. In a recent study, Aronowitz and Ellenhorn compared four different commercial cell separation systems – Cytori, Lipokit, PNC’s Multi Station (PNC. International, Gyeonggi- do, Republic of Korea) and CHA Biotech Cha-Station (CHA Biotech, Kangnamgu, Republic of Korea) – evaluating the cell yield, frequency of CD34^+^CD31^-^CD45^-^ and endothelial cells as well as colony-forming units of the isolated cells, showing a clear superiority for the Cytori system [18]. However, the residual enzyme levels observed with Lipokit was 57-fold higher than that observed with Cytori, and this can explain why in our work, in which we used not the SVF but the lipoaspirate enriched in the isolated cells, we did not observe such a clear difference between Cytori and Lipokit. In fact, addition of the isolated cells to the lipoaspirates could have neutralized the residual enzymatic activity, possibly reducing the negative effect of the enzymes on the isolated cells. This suggests the importance of the standardization of the sample to be used to define the properties of the donated fat graft. In Aronowitz and Ellenhorn’s work, moreover, the authors did not study ASCs and a clinical evaluation is missing.

Regarding the clinical results, in this pilot study we used an ultrasound evaluation of the subcutaneous thickness of the breast area corresponding to the SMQ and MMQ. The clinical evaluation of the results is a key point to establish the entity of the graft take and, ultimately, the effectiveness of the technique. In this regard, the magnetic resonance imaging and three-dimensional surface imaging are considered ideal examinations to quantify the entity of the graft takes. In fact, both techniques are non-invasive tools characterized by high accuracy and reproducibility in evaluating the effective volume persistence [53]. However, these methods are cost-effective, and, in the case of the three-dimensional surface imaging, no information on the breast tissue is available. To ensure the possibility to have easy, fast and low-cost measurements, we have employed an ultrasound device [54, 55]. The ultrasonographic analysis gives the possibility to extend the analysis to several patients and even to increase the frequency of the examinations, thus improving the evaluation of the fat graft persistence/resorption kinetic. In this paper we have shown that, 6 months after fat grafting, patients treated with stem cell-enriched lipoaspirates showed, with respect to those treated with the standard procedure, a significant superior gain in the subcutaneous thickness of both MMQ and SMQ; moreover, the significant superior thickness loss between 6 and 12 months in the MMQ of patients treated with standard lipoaspirates confirmed a better stability over time of fat grafts obtained with stem cell-enriched procedures.

The millimeter size of the thickness analyzed has made ultrasound imaging a proper, easy, and low-cost technique to be used for the measurements. Moreover, to overcome the subjective nature of ultrasound measurements, a dedicated team has been selected to control the patients and a standardization of the data collection procedure through a nine-quadrant breast pattern has been implemented.

Our results are in line with all reports indicating the clinical advantage of using the CAL procedure over a standard lipotransfer [17, 23, 39–42, 44].

Recently, Peltoniemi and colleagues raised doubts regarding the advantage of using stem cell enrichment in cosmetic fat transplantation to the breast [56]. Specifically, for breast augmentation they used either lipoaspirates obtained by water-assisted lipotransfer or water-assisted lipotransfer lipoaspirate enriched with stromal stem cells obtained using the Cytori system. The water-assisted lipotransfer method is based on the use of the Bodyjet System (Body-Jet; Human Med, Eclipse Ltd., Dallas, Texas), which allows a gentle liposuction through the aid of a water jet pulse dissecting the adipocytes from the subcutaneous tissue; its effectiveness, in terms of both fat graft survival rate and overall clinical outcome, has been reported by different groups [57, 58]. However, in Peltoniemi and colleagues’ work, no differences in the ASC phenotype was seen between the two lipoaspirates, thus suggesting that a more specific study on ASC presence and function in the two lipoaspirates would add crucial information.

Considering instead the three different devices, despite the fact that, accordingly with *in vitro* data, Cytori-treated patients displayed a trend to a better clinical outcome, no significant differences between the different CAL procedures could be detected, perhaps because of the small number of analyzed patients.

In fact, the major weakness points of this study are represented by the sample size of enrolled patients and by the short-term follow-up (12 months). A larger case study and a longer follow-up period are needed to confirm the results, and a double-blind ultrasound measurement study is needed to assess the accuracy and reproducibility of our standardized protocol. Moreover, a cost analysis on a large population would clarify whether CAL represents a real advantage. In fact, lipostructure techniques are widespread and usually patients must be subjected to repeated interventions to achieve the optimum result. The use of stem cells should increase the long-term graft performance, reducing the number of the surgical procedures required to reach the desired clinical outcome. The cost analysis should therefore take into account the costs related to the CAL procedures and the savings due to the reduction in the number of surgeries required.

## Conclusions

In this paper, applying our *in vitro* protocol to isolate, quantify and characterize SVF cells and ASCs from fat grafts, we have demonstrated a significant superiority of the enzymatic stem cell enrichment procedures over the standard procedure. Accordingly, our *in vivo* protocol, based on the ultrasonographic assessment of the subcutaneous thickness of the breast area, confirmed the clinical advantage of CAL techniques in reducing the graft resorption at 1 year, especially in the central part of the breast.

## Electronic supplementary material

Additional file 1: **Presents supplemental materials and methods.** (DOCX 14 KB)

Additional file 2: **Is Table S1 presenting the surface immunophenotype of ASCs isolated from the samples obtained with different procedures.** (DOCX 16 KB)

Additional file 3: **Is Figure S1 showing a comparison of lipoaspirates obtained with the standard procedure with those obtained from the Lipokit, Cytori and Fastem devices before stem cell enrichment, in terms of: cell yield, frequency of colony-forming unit cells and fraction of CD34** ^**+**^ **CD45** ^**–**^ **CD31** ^**–**^ **cells in the isolated SVF as well as the population doubling time of obtained ASCs.** Results are expressed as mean ± standard deviation. (TIFF 1 MB)

## Abbreviations

ASC: adipose stem cell
CAL: cell-assisted lipotransfer
Cytori: Cytori Celution System
Fastem: Fastem Corios
Lipokit: Lipokit Medikhan System
MMQ: median-median quadrant
SMQ: superior-medial quadrant
SVF: stromal vascular fraction.
