# Supplementary material for: Adipose tissue derived stem cells: in vitro and in vivo analysis of a standard and three commercially available cell-assisted lipotransfer techniques
Source: Stem Cell Res Ther. 2015 Jan 5;6(1):2. doi: 10.1186/scrt536 (PMC4417272; doi:10.1186/scrt536)
Supplement: Supplementary file 1 — Additional file 1: Presents supplemental materials and methods. (DOCX 14 KB) [file 13287_2014_418_MOESM1_ESM.docx]

Supplemental Materials and Methods

**Lipostructure techniques**

**Standard Technique**

The standard lipofilling technique performed in our department follows Coleman’s main indications. Briefly, after injecting a tumescent solution (1000ml of saline solution, 600 mg mepivacain, 1g adrenaline) in the selected donor area, lipoaspiration is performed with a 3mm blunt cannula and the lipoaspirate is collected in a drain bottle connected with a low-pressure system of aspiration (0.4 bar) and centrifuged for 1 minute at 3000g. Infiltration is conducted with 2.5 ml syringes with a smaller blunt cannula. The caliber, the length, the shape, the type of tip of the cannula is chosen according to the area to be treated.

**CYTORI CELUTION SYSTEM^®^**

The Cytori Celution^®^ 800/CRS System is a CE marked device that allows the withdrawal and isolation of ADSC from the patient and their use in the same session to the same patient in a single fat grafting procedure.

The Celution^®^ System is an automated, sterile and closed processing platform. The device is used together with the Celase^TM^ 835/CRS enzyme, a mixture of highly purified collagenases and proteases, to obtain the isolation of ADSC. During the fat grafting procedure, half of the lipoaspirates is used for the isolation of ADSC through the enzymatic digestion and the other half is washed and gravity filtered in the Celution^®^ System to create an aqueous fat graft.

**LIPOKIT MEDIKAN**

Lipokit is a CE marked and FDA approved device for autologous fat transfer. It is defined an all-in-one closed device, because it consists of a built in vacuum and pressure pump with air hose connection to the infiltration, suction or injection cannula through a designed Fat Processing Unit(FPU) collection syringe. The injection can be performed through the same syringe by air pressure or manually. A syringe, designed with a weight-mesh filter piston, is used in order to have a “squeeze” effect on the lipoaspirate during the centrifugation at 1200g per 3 min. Half of the lipoaspirate is processed immediately with centrifugation and the other half is treated with the Celltibator, a device designed for incubation and isolation of stem cell. The Roche Laboratories collagenase solution (Liberase) is then added for 40 minutes to part of the “lipo-condensed” fat in order to digest the cells. At the end of the process the stem cell components are mixed with the remaining condensed adipose tissue of the patient. The enriched adipose tissue is then grafted to the receiving area of the patient.

**FASTEM/CORIOS**

This technique enables the infiltration of minimally manipulated fat grafts enriched by stromal vascular fraction, obtained through the use of multiple manual filtering system. The FaStem kit (Fat Fast Stem Cells) is a concentrator of mesenchymal stem cells and stromal vascular fraction (SVF) from the adipose tissue. This device enables the surgeon to obtain a concentrated adipose lipoaspirate. This device is sterile and latex free and is composed by four separated envelopes, each one dedicated to a different step of the procedure: liposuction, adipose pre-treatment, separation and dressing. The most important devices for this procedure are a 250 ml bag with a 120 microns filter and teflon cylindrical insert, and a bag with three-way cock and prolongs pre-assembled.

After the liposuction, half of the lipoaspirates is treated with the standard technique adopted in our Departement and other half is kept for the concentration procedure. The syringe with the lipoaspirate is connected directly to the first filter bag (pre-treatment). The bag has to be kneaded and compressed until the lipoaspirates reach the bottom of the bag through the filter. From six to twelve 20 ml syringes are then filled with the first filtrated tissue and centrifuged at 400 gpm for 10 minutes. After the centrifugation, the lipoaspirates is ready to be divided through a three way connectors. The bottom level of the lipoaspirates is separated from the oil debris and collected in a 10 ml syringe. This procedure is then repeated for all the 20 ml syringes. At the end, the 10 ml syringe with the “ultrafiltrated” solution is added to the lipoaspirates normally treated, to obtain an enriched fat grafting, ready for the lipostructure procedure.
