# Supplementary material for: Adipose tissue derived stem cells: in vitro and in vivo analysis of a standard and three commercially available cell-assisted lipotransfer techniques
Source: Stem Cell Res Ther. 2015 Jan 5;6(1):2. doi: 10.1186/scrt536 (PMC4417272; doi:10.1186/scrt536)
Supplement: Supplementary file 2 — Additional file 2: Is Table S1 presenting the surface immunophenotype of ASCs isolated from the samples obtained with different procedures. (DOCX 16 KB) [file 13287_2014_418_MOESM2_ESM.docx]

**Table S1.** Surface immunophenotipe of ADSCs isolated from the samples obtained with different procedures.

|  | **LIPOKIT (n=5)** | | **CYTORI (n=5)** | | **FASTEM (n=5)** | | **STANDARD LIPOASPIRATE § (n=10)** |
| --- | --- | --- | --- | --- | --- | --- | --- |
|  | **NE** | **E** | **NE** | **E** | **NE** | **E** |  |
| **CD13** | **99.58±0.53** | **99.53±0.41** | **99.84±0.13** | **99.95±0.05** | **97.58±4.02** | **99.99±0.02** | **99,96±0,06** |
| **CD59** | **97.96±0.06** | **96.84±2.05** | **97.94±2.53** | **99.45±0.59** | **98.72±1.49** | **98.63±0.86** | **99,62±0,23** |
| **CD49d** | **94.88±1.24** | **96.65±1.68** | **90.69±6.48** | **97.31±1.89** | **98.68±0.56** | **99.26±0.28** | **98,93±1,10** |
| **CD73** | **99.62±0.28** | **99.07±0.38** | **99.00±1.09** | **99.86±0.15** | **99.50±0.20** | **99.81±0.12** | **99,56±0,36** |
| **CD90** | **97.23±3.87** | **99.4±0.72** | **99.43±0.22** | **99.00±0.44** | **99.43±0.83** | **99.22±0.64** | **99,80±0,24** |
| **CD44** | **99.67±0.21** | **83.31±22.74** | **99.84±1.32** | **99.85±0.10** | **99.24±0.48** | **99.72±0.10** | **94±8,73** |
| **CD29** | **59.18±23.99** | **49,11±12.76** | **34.99±23.72** | **51.29±29.23** | **84.08±9.05** | **66.16±17.32** | **62,47±32,7** |
| **CD105** | **99,90±0.05** | **97.22±2.70** | **98.55±0.89** | **99.44±0.58** | **98.54±1.29** | **99.08±1.24** | **74,50±34,21** |
| **CD271** | **1.05±0.86** | **2.40±2.37** | **5.66±6.35** | **3.02±3.16** | **3.72±2.15** | **2.91±2.57** | **3,19±2,65** |
| **CD49b** | **98.99±1.40** | **99.69±0.14** | **98.43±2.36** | **99.86±0.06** | **98.93±1.54** | **99.96±0.04** | **99,57±0,94** |
| **CD49a** | **58.19±1.15** | **20.47±18.41** | **38.45±19.99** | **39.53±28.51** | **62.98±19.08** | **52.18±14.27** | **54,31±31,24** |
| **KDR** | **38.06±0.20** | **44.10±60.31** | **6.98±9.15** | **26.48±29.16** | **51.71±23.11** | **52.11±60.39** | **1,70±2,51** |
| **CD66e** | **0.75±0.08** | **1.22±0.66** | **7.24±8.27** | **0.96±0.75** | **2.93±0.57** | **3.01±1.89** | **2,95±4,22** |
| **ABCG2** | **0.95±0.05** | **2.18±1.15** | **1.2±0.7** | **15.56±27.36** | **2.37±2** | **22.28±29.20** | **0,78±0,41** |
| **CD144** | **0.46±0.08** | **0.13±0.06** | **3.78±5.08** | **5.58±10.61** | **5.27±4.65** | **1.50±0.08** | **ND** |
| **CD10** | **3.95±4.71** | **3.55±2.91** | **27.33±11.38** | **15.34±13.94** | **28.00±25.85** | **16.96±24.14** | **50,80±28,21** |
| **CD117 (C-Kit)** | **1.57±1.46** | **12.34±4.69*** | **3.90±3.75** | **20.41±16.92*** | **2.12±1.52** | **7.46±12.37** | **0,15±0,23** |
| **CD133** | **0.08±0.11** | **0.19±0.12** | **0.34±0.32** | **0.15±0.10** | **0.23±0.15** | **0.23±0.02** | **0,27±0,14** |
| **CD34** | **0.07±0.02** | **0.01±0.01** | **0.51±0.58** | **0.02±0.02** | **0.02±0.03** | **0.09±0.09** | **0,24±0,69** |
| **HLA-DR** | **0.03±0.01** | **0.36±0.24** | **14.53±16.95** | **1.72±2.90** | **0.12±0.09** | **0.13±0.14** | **0,04±0,08** |
| **CD45** | **0.13±0.06** | **0.08±0.04** | **0.37±0.34** | **0.3±0.2** | **0.26±0.20** | **0.44±0.14** | **0,23±0,30** |

NE: not stem cell-enriched lipoaspirates; E: stem cell-enriched lipoaspirates; § Modified Coleman’s procedure; *, p<0.05 vs NE.
