# Supplementary figures and images for: Adipose tissue derived stem cells: in vitro and in vivo analysis of a standard and three commercially available cell-assisted lipotransfer techniques
Source: Stem Cell Res Ther. 2015 Jan 5;6(1):2. doi: 10.1186/scrt536 (PMC4417272; doi:10.1186/scrt536)

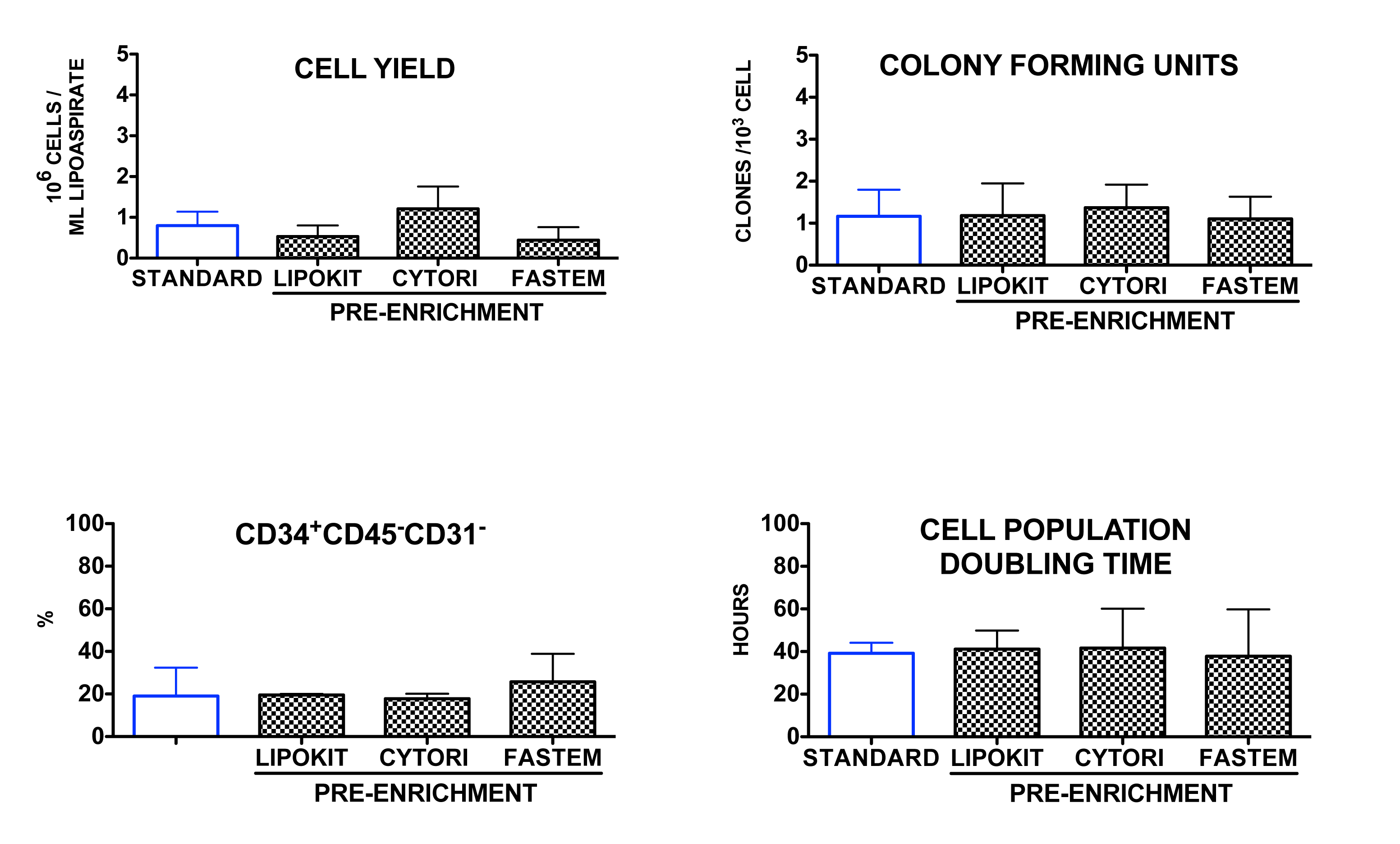

Supplement: Supplementary file 3 — Additional file 3: Is Figure S1 showing a comparison of lipoaspirates obtained with the standard procedure with those obtained from the Lipokit, Cytori and Fastem devices before stem cell enrichment, in terms of: cell yield, frequency of colony-forming unit cells and fraction of CD34 + CD45 – CD31 – cells in the isolated SVF as well as the population doubling time of obtained ASCs. Results are expressed as mean ± standard deviation. (TIFF 1 MB) [file 13287_2014_418_MOESM3_ESM.tiff]
